# Pathway and Network Analyses Identify Growth Factor Signaling and MMP9 as Potential Mediators of Mitochondrial Dysfunction in Severe COVID-19

**DOI:** 10.3390/ijms24032524

**Published:** 2023-01-28

**Authors:** Ya Wang, Klaus Schughart, Tiana Maria Pelaia, Tracy Chew, Karan Kim, Thomas Karvunidis, Ben Knippenberg, Sally Teoh, Amy L. Phu, Kirsty R. Short, Jonathan Iredell, Irani Thevarajan, Jennifer Audsley, Stephen Macdonald, Jonathon Burcham, PREDICT-19 Consortium, Benjamin Tang, Anthony McLean, Maryam Shojaei

**Affiliations:** 1 Department of Intensive Care Medicine, Nepean Hospital, Kingswood, NSW 2747, Australia; 2 Centre for Immunology and Allergy Research, The Westmead Institute for Medical Research, Sydney, NSW 2145, Australia; 3 Faculty of Medicine and Health, Sydney Medical School Nepean, Nepean Hospital, The University of Sydney, Kingswood, NSW 2747, Australia; 4 Department of Microbiology, Immunology and Biochemistry, University of Tennessee Health Science Center, Memphis, TN 38163, USA; 5 Institute of Virology Münster, University of Münster, 48149 Münster, Germany; 6 Sydney Informatics Hub, Core Research Facilities, The University of Sydney, Sydney NSW 2006, Australia; 7 Medical ICU, 1st Department of Internal Medicine, Charles University and Teaching Hospital Pilsen, 323 00 Plzeň, Czech Republic; 8 Department of Microbiology, St. George Hospital, Sydney, NSW 2217, Australia; 9 Research and Education Network, Western Sydney Local Health District, Westmead Hospital, CNR Darcy and Hawkesbury Roads, Sydney, NSW 2145, Australia; 10 Faculty of Medicine and Health, Sydney Medical School Westmead, Westmead Hospital, The University of Sydney, Sydney, NSW 2145, Australia; 11 School of Chemistry and Molecular Biosciences, The University of Queensland, Brisbane, QLD 4072, Australia; 12 Centre for Infectious Diseases and Microbiology, The Westmead Institute for Medical Research, Sydney, NSW 2145, Australia; 13 Faculty of Medicine and Health, School of Medical Sciences, The University of Sydney, Sydney, NSW 2145, Australia; 14 Westmead Hospital, Western Sydney Local Health District, Sydney, NSW 2145, Australia; 15 Sydney Institute for Infectious Disease, The University of Sydney, Sydney, NSW 2145, Australia; 16 Victorian Infectious Disease Service, The Royal Melbourne Hospital at the Peter Doherty Institute for Infection and Immunity, Melbourne, VIC 3050, Australia; 17 Department of Infectious Diseases, The University of Melbourne at the Peter Doherty Institute for Infection and Immunity, Melbourne, VIC 3000, Australia; 18 Centre for Clinical Research in Emergency Medicine, Harry Perkins Institute of Medical Research, Royal Perth Hospital, Perth, WA 6000, Australia; 19 Medical School, University of Western Australia, Perth, WA 6009, Australia; 20 Emergency Department, Royal Perth Hospital, Perth, WA 6000, Australia; 21 Centre for Clinical Research in Emergency Medicine, Royal Perth Bentley Group, Perth, WA 6000, Australia

**Keywords:** COVID-19, metabolism, endocrine, MMP9, RNA sequencing, DEG, Metacore

## Abstract

Patients with preexisting metabolic disorders such as diabetes are at a higher risk of developing severe coronavirus disease 2019 (COVID-19). Mitochondrion, the very organelle that controls cellular metabolism, holds the key to understanding disease progression at the cellular level. Our current study aimed to understand how cellular metabolism contributes to COVID-19 outcomes. Metacore pathway enrichment analyses on differentially expressed genes (encoded by both mitochondrial and nuclear deoxyribonucleic acid (DNA)) involved in cellular metabolism, regulation of mitochondrial respiration and organization, and apoptosis, was performed on RNA sequencing (RNASeq) data from blood samples collected from healthy controls and patients with mild/moderate or severe COVID-19. Genes from the enriched pathways were analyzed by network analysis to uncover interactions among them and up- or downstream genes within each pathway. Compared to the mild/moderate COVID-19, the upregulation of a myriad of growth factor and cell cycle signaling pathways, with concomitant downregulation of interferon signaling pathways, were observed in the severe group. Matrix metallopeptidase 9 (*MMP9)* was found in five of the top 10 upregulated pathways, indicating its potential as therapeutic target against COVID-19. In summary, our data demonstrates aberrant activation of endocrine signaling in severe COVID-19, and its implication in immune and metabolic dysfunction.

## 1. Introduction

The COVID-19 pandemic has been a global health concern since December 2019. Patients with COVID-19 show a wide spectrum of disease manifestations with the majority experiencing mild/moderate symptoms including fever, cough, fatigue, and muscle pain. Around 14–20% develop a severe to critical illness, associated with adverse outcomes and higher mortality [1]. Immune dysregulation is evident in severe COVID-19, characterized by impaired type I interferon (IFN-I) response, aberrant activation of neutrophils, lymphopenia, and overproduction of proinflammatory cytokines [2,3,4,5,6,7,8,9]. Immunotherapies including corticosteroids (e.g., dexamethasone), kinase inhibitors (e.g., baricitinib), interleukin 1 receptor (IL-1R) antagonist/antibody anakinra, interleukin 6 receptor (IL-6R) antagonist/antibody tocilizumab and sarilumab have shown some beneficial effects in the selected group of patients [10]. However, treatment for severe COVID-19 is still limited due to the complexity of disease pathogenesis and heterogeneity in the patients’ immune status. Discovery of novel therapeutics targeting the underlying cause of immune dysregulation would offer additional and perhaps more effective treatments, which is essential for alleviating the disease burden of COVID-19. 

The host immune response is tightly linked to the body’s metabolic status. Immune dysregulation is often associated with an underlying metabolic dysfunction and vice versa [11,12,13,14]. It has been established that preexisting metabolic disorders such as diabetes mellitus, obesity, hypertension, or cardiovascular disease, are strong risk factors for developing severe COVID-19 [15,16]. Investigating the interaction between immune and metabolic pathways would shed some light on the mechanisms underlying disease progression. Metabolism is known also to be regulated by the endocrine system, through releases of various growth factors [17]. One such example is the regulation of glucose metabolism by insulin [18]. Other growth factors such as insulin-like growth factor 1 (IGF1) and hepatocyte growth factor (HGF) also play a role in regulating glucose metabolism [19,20]. Insulin, IGF1, and HGF have also been implicated in regulating immune response in diabetes and cancer [21,22,23]. 

At the cellular level, metabolism is controlled by mitochondria, which supplies energy by converting carbohydrates, lipids, and proteins into adenosine triphosphate (ATP) via oxidative phosphorylation (OXPHOS). Energy produced by mitochondria is used to support the synthesis of macromolecules that are essential for cell growth and proliferation. In addition to energy production, mitochondrion also plays pivotal roles in cell cycle regulation [24] and apoptosis [25,26]. Furthermore, mitochondrion is a signaling organelle that mediates innate immune responses, leading to production of IFN-I and pro-inflammatory cytokines [27,28,29]. The health of mitochondrion is therefore crucial for maintaining a healthy immunity. Notably, patients with mitochondrial diseases often suffer from recurrent infections, an indication of underlying immune dysregulation [30,31]. Under physiological conditions, the health and function of mitochondrion is maintained through a highly regulated cycle consisting of mitochondrial dynamics (fusion and fission), mitochondrial biogenesis (synthesis of new mitochondria), and mitophagy (removal of dysfunctional mitochondria via autophagy) [32]. Balance between mitochondrial biogenesis and mitophagy is crucial for maintaining the status quo (mitochondrial homeostasis) and mitochondrial function thereof. Many signaling pathways are involved in the regulation of mitochondrial biogenesis and homeostasis, one of which is the IGF1 signaling pathway [33,34]. Mitochondrial homeostasis is perturbed under stress or an inflammatory condition, leading to mitochondrial dysfunction. Inflammatory cytokines (e.g., tumor necrosis factor (TNF), interleukin 1 β (IL-1β), and interferon γ (IFN-γ)) and oxidative stress (e.g., reactive oxygen species (ROS) and nitric oxide (NO)), can induce mitochondrial biogenesis, perhaps as part of compensatory stress response, which may lead to accumulation of damaged mitochondria. Nuclear factor kappa-light-chain-enhancer of activated B cells (NF-κB), mitogen-activated protein kinase (MAPK), and protein kinase B (PKB)/Akt-dependent signaling pathways mediate the activation of mitochondrial biogenesis induced by proinflammatory cytokines and oxidative stress [35]. Mitochondrial homeostasis can also be disrupted by viral infection as a mechanism to evade host immunity [36]. It has been shown that severe acute respiratory syndrome coronavirus 2 (SARS-CoV-2) RNA or protein can directly interact with the host mitochondria or nucleolus [37,38,39] and downregulate genes that are associated with mitochondrial dynamics and respiration [40,41]. However, it is not clear how mitochondrion and its regulation, vary with differing disease severities and hence contribute to disease outcomes in COVID-19. Our current study is aimed to address this gap, which is crucial for deciphering the key mechanism underlying COVID-19 progression. Our data demonstrates the role of growth factor signaling in mediating immune and metabolism interactions in severe COVID-19.

## 2. Results

### 2.1. Description of Human Cohort

Demographic and clinical characteristics of participants are summarized in Table 1. Participants were divided into two groups: mild/moderate (MldMod) (World Health Organization (WHO) severity levels 2–5), and severe (Svre) (WHO severity levels 6–9). Sex proportion for the two groups were: 38 (64%) males for the MldMod group, and 18 (62%) males for the Svre group. Median age for the two groups were: 64 years (interquartile range (IQR): 49.5–76.5) for the MldMod group, and 59 years (IQR: 50.0–69.0) for the Svre group. There were no significant differences in age and sex ratio between MldMod and Svre group. All subjects from MldMod and Svre groups (*n* = 88) were hospitalized. Mean length of hospital stay was 17 days for the MldMod group, and 27 days for the Svre group. Twelve (20%) subjects from the MldMod group were admitted to the intensive care unit (ICU). Fifteen (52%) subjects from the Svre group were admitted to ICU with a longer length of stay (mean of 17 days). Mortality rate was higher in the Svre group (41.5%) compared to the MldMod (13.5%). Seventy-one healthy volunteers were included as healthy controls (HC). Median age of the healthy controls was 50 years (IQR: 44.25–54, with 50:50 sex ratio).

### 2.2. Principal Component Analysis Reveals Overall Differences between Mild/Moderate and Severe COVID-19

**Figure 1:**
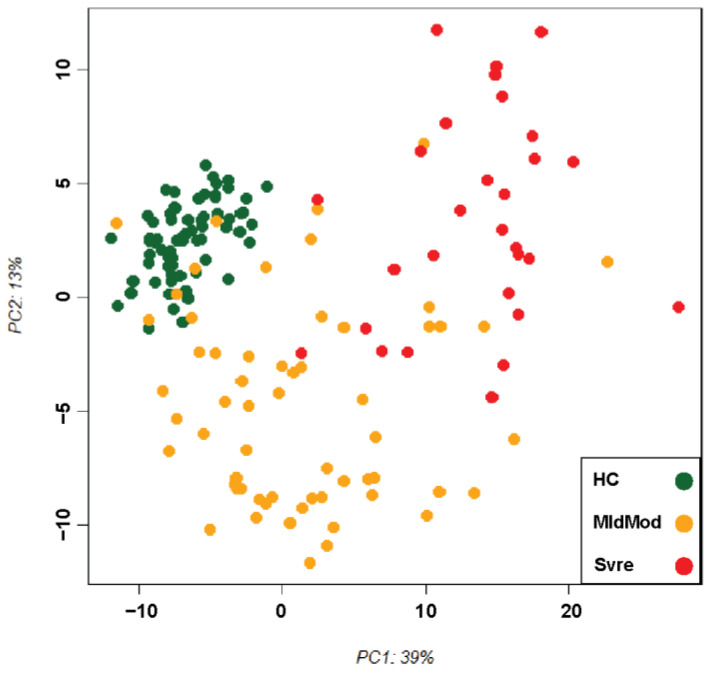
Principal component analysis (PCA) of all samples. PCA plot of PC1 and PC2 is based on rlog transformed (DESeq2) normalized expression values. It shows levels of healthy controls (HC), mild/moderate (MldMod), and severe (Svre) COVID-19 cases by different colours.

Principal component analysis (PCA) was performed to examine the overall variance in genes related to mitochondrial functions (as listed in Supplement Datasheet S1), which included a total of 1623 unique mitochondria-encoded, or nucleus-encoded mitochondrial genes extracted from all the gene sets listed in Supplement Datasheet S2. PCA plot showed a good separation between healthy controls (HC) and COVID-19 (COVID) and a clear trend from mild/moderate to severe (Figure 1). Similar separation among the groups was also observed when all 19,220 coding genes were used for the PCA (Supplementary Figure S1). Differences among the groups were also reflected by the change in cellular composition (by performing a deconvolution analysis of our bulk RNAseq data), with increased neutrophil and endothelial cell populations associated with the more severe group (Supplementary Figure S2).

### 2.3. Differential Expression Gene (DEG) Analysis Reveals Differences between Mild/Moderate and Severe COVID-19, Each of Which Is Associated with a Unique Set of Differentially Expressed Mitochondrial Genes

**Figure 2:**
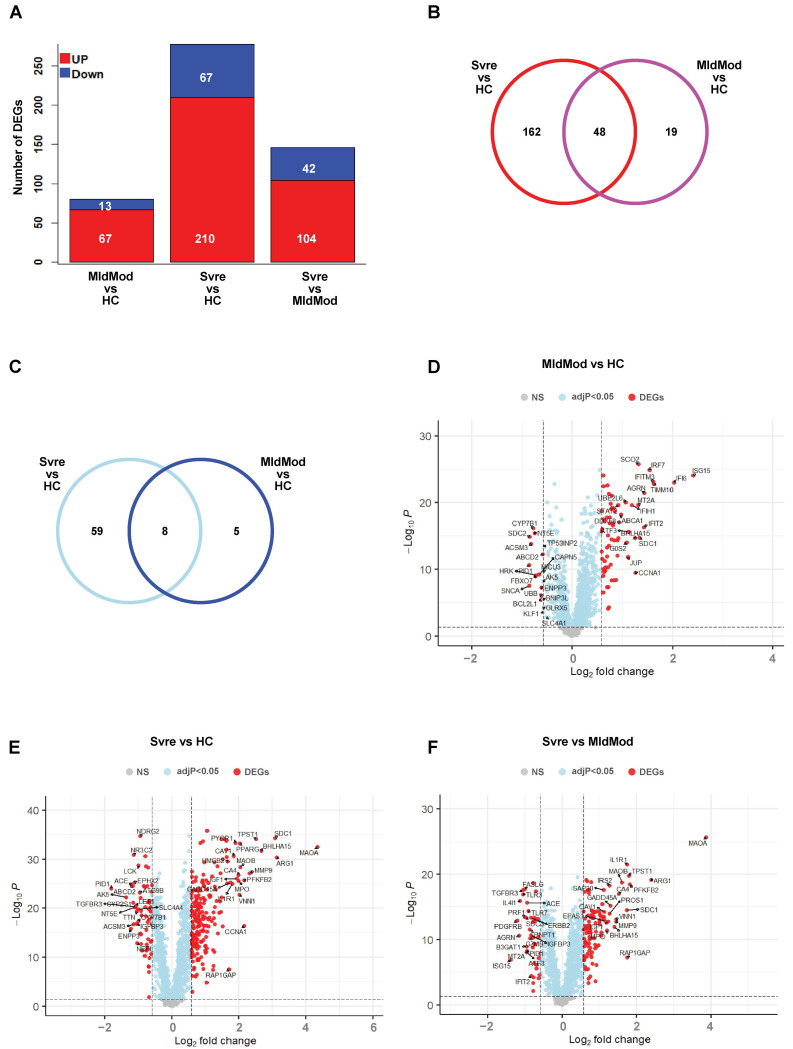
DEGs from comparisons of severity groups. (**A**) Bar diagram showing the number of up- (in blue) and downregulated (in red) DEGs from three comparisons: mild/moderate versus healthy controls (MldMod vs. HC); severe versus healthy control (Svre vs HC), and severe versus mild/moderate (Svre vs. MldMod). Y axis: number of DEGs. (**B**) Venn diagram showing the number of overlapping or nonoverlapping upregulated DEGs between comparison MldMod vs. HC (purple) and comparison Svre vs. HC (red). (**C**) Venn diagram showing the number of overlapping or nonoverlapping downregulated DEGs between comparison MldMod vs. HC (dark blue) and comparison Svre vs. HC (light blue). (**D**–**F**) Volcano plots showing the significantly up- or downregulated DEGs (red) (with adjusted *p*-value of <0.05 and an absolute 1.5-fold ([log_2_] > 0.58) difference in expression levels) in the three comparisons as in (**A**), respectively. Name of the top 20 significantly up- or downregulated DEGs were shown.

To determine how SARS-CoV-2 infection impacts mitochondrial function or regulation in patients with mild/moderate or severe COVID-19, we performed differential expression gene (DEG) analysis on the selected genes (*n* = 1623 genes) (Supplemental Datasheet S2). When compared to healthy controls, there were 67 up- and 13 downregulated DEGs in the mild/moderate group and 210 up- and 67 downregulated DEGs in the severe group (Figure 2A). There were 104 up- and 42 downregulated DEGs in the severe group compared to the mild/moderate one. Figure 2B,C show the unique or overlapping up- or downregulated DEGs in the severe or the mild/moderate group compared to the healthy control, respectively. Figure 2D–F, show the top 20 up- or downregulated genes for each comparison group respectively, namely mild/moderate vs. healthy control, severe vs. healthy control, and severe vs. mild/moderate. 

### 2.4. Pathway Enrichment Analysis Reveals Regulation or Dysregulation of Pathways Associated with Mild/Moderate or Severe COVID-19

**Figure 3:**
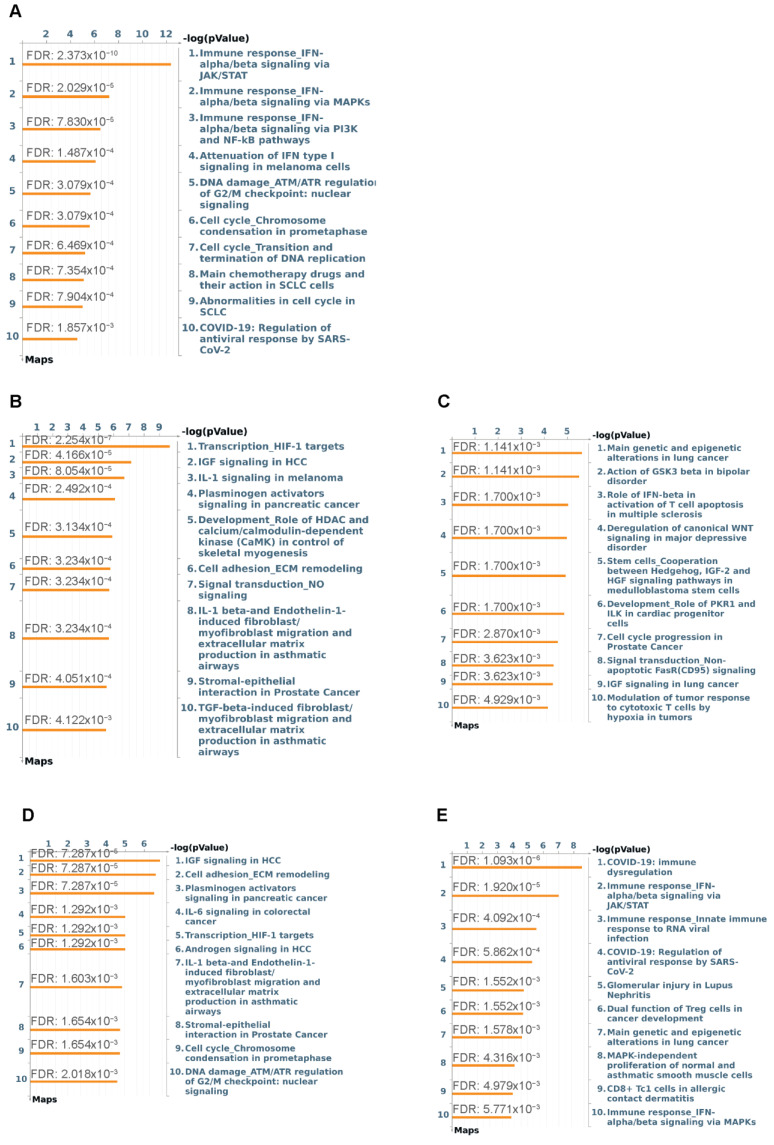
Metacore pathway enrichment analysis of DEGs. (**A**) Top 10 most significantly enriched pathways map for the upregulated DEGs comparing mild/moderate to healthy control (MldMod vs. HC_UP). (**B**) Top 10 most significantly enriched pathways map for the upregulated DEGs comparing severe to healthy control (Svre vs. HC_UP). (**C**) Top 10 most significantly enriched pathways map for the downregulated DEGs comparing severe vs. healthy control (Svre vs. HC_DOWN). (**D**) Top 10 most significantly enriched pathways map for the upregulated DEGs comparing severe to mild/moderate (Svre vs. MldMod_UP). (**E**) Top 10 most significantly enriched pathways map for the downregulated DEGs comparing severe to mild/moderate (Svre vs. MldMod_DOWN). False discovery rate (FDR) for each enriched pathway was shown.

To determine which pathways were significantly altered by COVID-19 and to elucidate differences between the mild/moderate and the severe cases, we performed pathway enrichment analysis by using all the DEGs as shown in Figure 2A. The top 10 most significantly enriched pathways for the upregulated DEGs in the mild/moderate COVID-19 group (vs. healthy control), are shown in Figure 3A. These pathways are mainly involved in type I IFN (IFN-α/β) antiviral signaling and cell cycle regulation including regulation of G2/M checkpoint and chromosome condensation. No significantly enriched pathways were associated with the downregulated DEGs Figure 3B,C respectively show the top 10 most significantly enriched pathways for the up- or downregulated DEGs in the severe COVID-19 (vs. healthy control). Notably, hypoxia response (transcription of hypoxia-inducible factor 1 (HIF-1) targets), endocrine signaling (IGF1 signaling), and proinflammatory cytokine signaling (interleukin 1 (IL-1)) were enriched in the severe group for the upregulated DEGs (Figure 3B). On the other hand, glycogen synthase kinase-3β (GSK-3β), prokineticin receptor 1 (PKR1) and Wnt signaling, were significantly enriched in the severe group for the downregulated DEGs (Figure 3C). Figure 3D,E respectively show the top 10 most significantly enriched pathways for the up- or downregulated DEGs in the severe COVID-19 (vs. mild/moderate). Endocrine (e.g., IGF1 and androgen) signaling pathways, proinflammatory cytokines (e.g., interleukin 6 (IL-6) and IL-1) signaling pathways, and hypoxia response (transcription of HIF-1 targets) were significantly enriched in the severe group for the upregulated DEGs (Figure 3D). As for the downregulated DEGs, the significantly enriched pathways were mainly involved in type I IFN immune response, and CD8+ T cells response (Figure 3E). Functional analyses of the gene ontology (GO) term enrichment for the DEGs were also performed to demonstrate the alterations of mitochondria-related cellular processes (Supplementary Figure S3).

### 2.5. Network Analysis Identifies That Severe COVID-19 Is Associated with Aberrant Activation of Endocrine Signaling Pathways Leading up to Upregulation of MMP9 and Downregulation of Innate Immune Signaling Pathways

**Figure 4:**
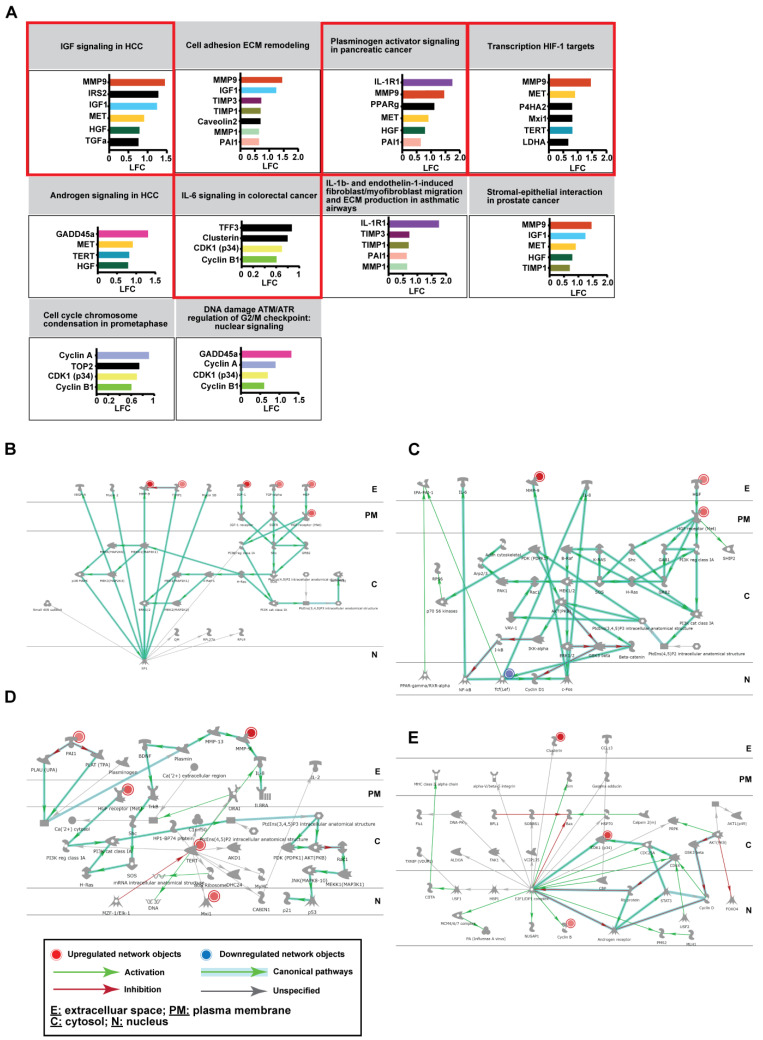

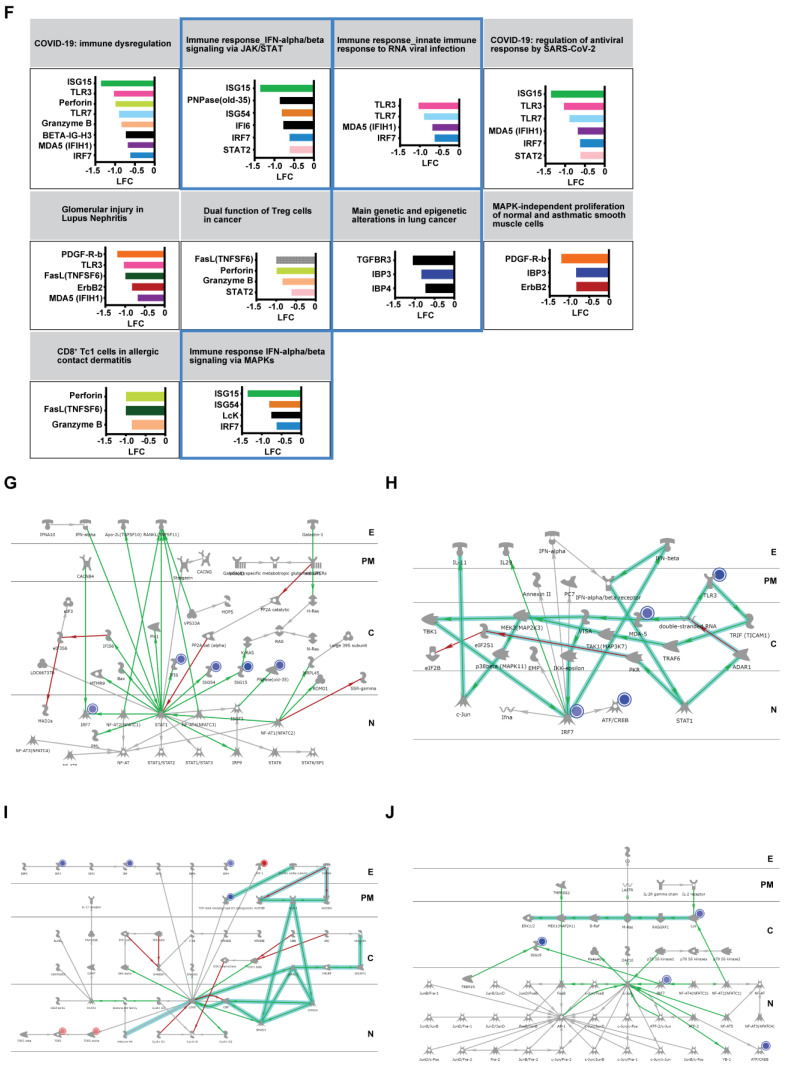
Metacore network analysis of genes from the enriched pathways. (**A**) Bar diagram showing Log_2_ fold change (LFC) of the genes that contribute to enrichment of the top 10 pathways from Figure 3D (Svre vs. MldMod_UP). Network analysis of the genes from four of the ten pathways (highlighted in red) were shown in (**B**) IGF1 signaling in HCC, (**C**) plasminogen activators signaling in pancreatic cancer, (**D**) transcription HIF-1 targets, (**E**) IL-6 signaling in colorectal cancer. (**F**) Bar diagram showing Log_2_ fold change (LFC) of the genes that contribute to enrichment of the top 10 pathways from Figure 3E (Svre vs. MldMod_DOWN). Network analysis of the genes from four of the ten pathways (highlighted in blue) were shown in (**G**) IFNa/b signaling via JAK/STAT, (**H**) innate immune response to RNA viral infection, (**I**) main genetic and epigenetic alterations, and (**J**) IFNa/b signaling via MAPKs. Red and blue circles denote upregulated and downregulated genes from the respective enriched pathway. The coloured solid line with arrows represents activation (green), inhibition (red) and unspecified (grey) effects between the two genes. Bold light green lines represent well-known canonical pathways. Abbreviations: E, extracellular space; PM, plasma membrane; C, cytosol; N, nucleus.

To further understand the pathways enriched in severe COVID-19 participants (compared to mild/moderate participants), we looked at the interactions among the genes within each of the top 10 significantly enriched pathways. Figure 4A,F, show the up- or downregulated genes (known as network objects) respectively, which contribute to the pathway enrichment. The majority of these genes (colour coded) were involved in multiple pathways, whereas a few (in black) were found only in one pathway. These genes were subjected to network analysis in Metacore. Networks and associated pathways entailing gene–gene interactions, are shown for four selected pathways. Figure 4B–E are for the upregulated pathways highlighted in red (Figure 4A) and Figure 4G–J are for the downregulated pathways highlighted in blue (Figure 4F). Networks and associated pathways for the other six pathways are provided in Supplemental Figure S4. Figure 4B,C show activation of *IGF1, HGF, TGF* signaling pathways, which lead to increased *MMP9* expression in the severe COVID-19 (versus mild/moderate). *MMP9* was found in five out of the ten enriched pathways, namely *IGF1* signaling in hepatocellular carcinoma (HCC), cell adhesion extracellular matrix (ECM) remodeling, plasminogen activators signaling in pancreatic cancer, transcription HIF-1 targets, and stromal-epithelial interaction in prostate cancer. Figure 4D show signaling pathway leading to increased cyclin-dependent kinase 1 (CDK1)/cycling B1 production in the severe COVID-19 (versus mild/moderate). Figure 4E–J show downregulation of IFNα/β response via the Janus kinas-signal transducer and activator of transcription (JAK/STAT) and MAPK pathways, which lead to decreased toll-like receptor 7 (*TLR7),* interferon induced with helicase C domain 1 *(IFIH1),* interferon regulatory factor 7 (*IRF7),* interferon-stimulated gene 15 *(ISG15),* interferon-induced protein with tetratricopeptide repeats 2 (*ISG54/IFIT2)*, and interferon-α inducible protein 6 (*IFI6)*. Subcellular localization of the network objects (as in Figure 4A,F) demonstrated that some were found in the mitochondria but majorities of them were not (Supplementary Datasheet S3).

## 3. Discussion

The importance of mitochondrion in COVID-19 pathogenesis and host mitochondrial transcriptome have been investigated in previous studies [39,40,41]. However, the role of mitochondrion, and its regulation in disease progression, are not well-studied. Given the fundamental involvement of mitochondrion in cell life, function, stress response and death, it is likely that mitochondrial health of the immune cells, is associated with disease outcome following SARS-CoV-2 infection [42]. Our current study seeks to better understand the host immune response from the aspect of cellular metabolism, regulation of mitochondrial respiration and organization, by appreciating the difference between mild/moderate and severe infections, identifying what separates mild/moderate and severe infections, and pinpointing genes or pathways that might be crucial for disease progression. Findings from our study would potentially help to understand the role of mitochondrion in regulating functions of tissue-specific immune cells and therefore aid the design of novel COVID-19 treatment, targeting bioenergetic dysfunction of the immune cells.

We analysed and compared genes specifically related to cellular metabolism, regulation of mitochondrial respiration and organization, and apoptosis, which were chosen to reflect the major functions and regulations of mitochondrion within the cell. Our data revealed distinct differences between mild/moderate and severe COVID-19, in terms of the number and types of differentially expressed genes (DEGs) when comparing each of them to the healthy control as well as when comparing between them. Severe COVID-19 encounters more alterations to the mitochondrion-related transcriptome than mild/moderate one, as evidenced by more up- or downregulated genes identified in the severe than in the mild/moderate COVID-19 when compared to the healthy control. Further differences were revealed by the DEGs identified in severe COVID-19 compared to mild/moderate participants. 

We then sought to determine what pathways these DEGs were associated with, to gain better understanding of their functions and roles in the disease. Pathway enrichment analysis revealed unique pathways for the DEGs identified in mild/moderate or severe COVID-19. In keeping with previous findings, we observed downregulated IFN-I response pathways in the severe COVID-19 compared to mild/moderate one [2,3,4]. Mitochondrion is known to mediate IFN-1 response through mitochondrial antiviral signaling (MAVS) and interferon regulatory factor-3 and -7 (IRF3/IRF7) [27,28]. Our results confirm the important role of mitochondrion in mediating innate immune response towards SARS-CoV-2, and perturbation to the mitochondrial aspect of this response occurs in severe cases of COVID-19. Concomitant with a downregulated IFN-I response, we observed an upregulation in hypoxia-inducing factor 1 (HIF1) transcription targets and *IGF1* signaling pathways. IGF1 is known for its role in regulating glucose metabolism [20]. Elevated glucose and glycolysis (hyperglycolysis) are observed in severe COVID-19 as a mechanism to promote viral replication, through ROS stabilization of HIF1α [43,44]. Hence, upregulated *IGF1* signaling could be a host response toward elevated glucose in severe COVID-19. IGF-1 has also been associated with promoting proinflammatory activity in human peripheral white blood cells [21].

We delved further into the pathways enriched in severe COVID-19 (compared to mild/moderate cases) by network analysis to look for connections among the genes within the pathway and interconnections among the pathways. Out of the top 10 significantly upregulated pathways in the severe cases, *MMP9* was found in five of them. Its upregulation is associated with *IGF1, HGF*, and *TGF* signaling through MAPK pathway. MMP9 belongs to the family of zinc-dependent endopeptidases, which plays important roles in cell proliferation, apoptosis, migration, and differentiation [45]. Overexpression of MMP9 in alveolar macrophages, bronchial tissues, sputum, and serum, has been previously associated with chronic obstructive pulmonary disease (COPD), emphysema, and asthma [46] and upregulation of *MMP9* in the blood has been associated with severe COVID-19 [7,47]. Serum level of MMP9 is found to be significantly elevated in the more severe cases of COVID-19 and in combination with brain-derived neurotrophic factor (BDNF), MMP9 has shown potential as a predictive marker for COVID-19 severity [47]. It is known that overproduction of MMPs leads to excessive tissue damage, neutrophil influx, neutrophil activation, and overproduction of proinflammatory cytokine IL-1β, hence contributing to the disease progression [7,48]. Our current study, for the first time, provides evidence on MMP9′s involvement in regulation of mitochondria-related functions and COVID-19 disease progression. Previously, MMPs have been implicated in mitochondrial dysfunction in diabetic retinopathy and cardiac disease. MMP9 in particular can be localized to mitochondria and induces mitochondrial dysfunction [49,50,51,52]. Our current study further supports the pivotal role of MMP9 in modulating immunometabolism via endocrine signaling pathways and could be a potential therapeutic target against severe COVID-19 [46]. 

We are aware of a major limitation of the current study. Larger numbers of participants in the severe COVID-19 group would add additional information on age and sex effects. Future study with the use of publicly available data would be useful to address this. Another limitation of the current study is that we are not able to discern if upregulated expression of *MMP9* and its associated signaling pathways in the severe COVID-19, is because of increased neutrophil population or cell-specific increased expression. Future analysis of single cell transcriptomic data would provide better understanding in this regard. 

In conclusion, through pathway and network analyses, our current study demonstrates an aberrant activation of endocrine (e.g., IGF1 and HGF) signaling pathways in the severe cases, as well as their connection to a upregulation of *MMP9*, a gene that has been implicated in the aberrant activation of neutrophil in severe COVID-19 [7] and mitochondrial dysfunction in diabetic retinopathy and cardiac disease [49,50,51,52]. Our study provides insight on the interconnection between immunometabolism and growth factor signaling and their roles in disease progression.

## 4. Materials and Methods

### 4.1. Study Design and Participants of Human Cohorts

In this study, 88 patients were recruited from multiple centers from Sydney, Melbourne, and Perth in Australia and a single center in Czech Republic between February 2020 and February 2021. The inclusion and exclusion criteria are as follows:

Inclusion criteria: (1) age ≥ 18 years old, (2) World Health Organization definition of influenza-like illness (fever of 38 °C or higher, cough, sore throat, nasal congestion, and illness onset within the last 10 days), and (3) patient with SARS-CoV-2 infection confirmed by virological testing respiratory samples (nasal/throat swab/sputum/bronchoalveolar lavage) collected from patients and tested for SARS-CoV-2 virus. All eligible patients were assessed by an admitting physician for likelihood of infection. Patients with a high likelihood of infection, based on history and clinical features, were also enrolled into the study.

Exclusion criteria: age < 18 years old.

Samples from 71 healthy volunteers included in this study were all collected prior to 2018. Study data were collected and managed by using REDCap electronic data capture tools [53,54] hosted at the University of Sydney.

### 4.2. Blood Sample Collection and RNA Isolation

Two and half millilitres of blood were collected into PAXgene Blood RNA tubes (Qiagen, Venlo, The Netherlands) from participants according to the manufacturer’s supplied protocol, resulting in a total of 203 samples (multiple samples were taken from some patients). Collected samples were inverted 8–10 times gently, immediately after blood collection, kept for ~2 h at room temperature, followed by incubation at −20 °C for 24 h. Thereafter tubes were transferred to −80 °C prior to processing. Total RNA was isolated from whole blood samples stored and stabilized in PAXgene RNA tubes according to the manufacturer’s guidelines (PreAnalytiX, Zurich, Switzerland). The quality and quantity of extracted RNA was evaluated by visualization of 28S and 18S band integrity on a Tapestation 4200 system (Agilent, Santa Clara, California, CA, USA) and stored at −80 °C.

### 4.3. Library Preparation and RNASeq

Libraries were prepared with 300 ng of total RNA per sample by using the Illumina Stranded Total RNA Prep with Ribo-Zero Plus (RZP) as per manufacturer instructions (Illumina, San Diego, CA, USA). Final libraries were cleaned by using beads (Beckman Coulter, Brea, CA, USA), quantified, and normalised with qPCR using NEBNext Library Quant Kit for Illumina. All libraries were pooled with 32 samples per lane and sequenced with 150 bp paired-end (PE) reads by using an Illumina NovaSeq 6000 with v1.5 chemistry and S4-300 flow cell. A minimum sequencing depth of 48.3 million (M) read pairs were generated from each library. Base calling and FASTQ conversion were complete with NovaSeq Control Software (NCS) v1.7.5, Real Time Analysis (RTA) v3.4.4 and Illumina DRAGEN BCL Convert 07.021.624.3.10.8. FASTQ files were uploaded into Partek Flow software (Partek Inc., Chesterfield, MO, USA), and primary QC was performed.

### 4.4. Mitochondrial Gene Sets

Forty-seven gene sets consisted of 7 Hallmark, 33 GOBP, 1 GOMF, 1 WP, 1 HP, and 4 REACTOME gene sets as listed in Supplement Datasheet S2 were obtained from the molecular signature database (Msigdb) (https://www.gsea-msigdb.org/gsea/msigdb/index.jsp, accessed on 29 September 2022) [55]. These gene sets are associated with the metabolic pathways, functional or structural maintenance of the mitochondrion, and mitochondrion-mediated immune response towards SARS-CoV2. A unique gene list generated from the abovementioned gene sets is listed in Supplement Datasheet S1.

### 4.5. Bioinformatic Analysis of RNASeq Data

FASTQ files containing raw sequencing data were quality controlled and preprocessed into analysis-ready count data by using the highly scalable RNASeq-DE workflow, available online at https://github.com/Sydney-Informatics-Hub/RNASeq-DE (v1.0.0), accessed on 10 December 2022 [56]. Default settings were applied unless otherwise described here. Briefly, 3′ adapter and polyA tails were trimmed from raw sequence reads with BBDuk (v37.89) [57]. An average of 89.2 million trimmed reads per sample were remaining. FastQC (v0.11.7) [58] was used to confirm that median sequence and base qualities scored Phred > 20. Quality checked, trimmed reads were aligned as pairs to the human reference genome, GRCh38 primary assembly and gene set release 106 (obtained from Ensembl) with STAR, setting –sjdbOverhand to 149. Sequencing batch level binary alignment (BAM) files were merged and indexed with SAMtools (v1.10) [59] to obtain sample level BAMs. HTSeq-count (v0.12.4) [60] with -s reverse was used to obtain feature level raw counts. Raw counts were annotated by using the package biomart (version 2.42.1, [61] by using function:

useEnsembl (biomart=“ensembl”, dataset=“hsapiens_gene_ensembl”, GRCh=38). Entries with no gene symbol were deleted. Then raw counts were normalized and log_2_ transformed by using function rlogTransformation from the DESeq2 package (version 1.16.1, [62]). An increment was added to the normalized values to make all values positive. For this analysis, mitochondrial genes were selected from different databases (Supplement Datasheet S1). Subsequently, identification of differentially expressed mitochondrial genes (DEGs), package LIMMA (version limma_3.42.2, [63,64]) was used with function model.matrix (~0 + group). Volcano plots were generated with the package EnhancedVolcano, version 1.8.0 [65]. Further analysis and visualization of expression data was performed by using the R software package (version 3.4.0) [66].

### 4.6. Metcore Pathway Enrichment and Network Analysis

To comprehensively dissect the pathways associated with the DEGs, MetacoreTM, a Cortellis Solution software (Clarivate Analytics, UK, https://clarivate.com/products/metacore/, accessed on 1 December 2022), was used to perform curated pathway-enrichment analysis and GO analysis. Comparing different phenotypes, three lists of differentially expressed mitochondrial related genes were generated and uploaded in MetaCore pathway analysis:

I. mild/moderate COVID-19 vs. healthy controls,
II. severe COVID-19 vs. healthy controls, and
III. mild/moderate vs. severe COVID-19.

Pathway enrichment analysis was used for analysing experimental data in terms of their enrichment of pathway maps. Pathway maps tool was used to identify the enriched pathways involving DEGs in terms of the hypergeometric distribution, and the *p*-values were calculated by using the default database as the background (based on a false discovery rate (FDR) *p* < 0.005). Analysis between the three comparison groups were based on an adjusted *p*-value of <0.05 and an absolute 1.5-fold ([log_2_] > 0.58) difference in expression levels. Changes in expression levels were presented as fold changes for a given gene. (FDR) adjustment was applied for multiple testing. An FDR of 5% was used as the cutoff to determine whether a pathway was statistically overrepresented in the gene list. Adjusted *p*-value are expressed in -log(*p*-value) and ranked by statistical significance. Genes denoted as network objects, were used to build network. The “Analyze network” building algorithm with number of nodes in a network “50” was used for analysis. Canonical pathways were chosen. The network with the highest number of total nodes was chosen.

## Figures and Tables

**Table 1:** Demographic and clinical characteristics of participants.

	HC (*n* = 71)	MldMod (WHO 2–5) (*n* = 59)	Svre (WHO 6–9) (*n* = 29)	*p* Values_ HC vs. MldMod	*p* Values_ HC vs. Svre	*p* Values_ MldMod vs. Svre
Sex (males/females)	36M/35F	38M/21F	18M/11F	ns	ns	ns
Age/years (median; IQR)	50 (IQR:44.25–54)	64 (IQR: 49.5–76.5)	59 (IQR: 50.0–69.0)	<0.0001	<0.05	ns
day_past_on_sympt	N/A	6	7	N/A	N/A	ns
**Outcome**						
LOS in hospital (days)	N/A	17	27	N/A	N/A	<0.0001
Admission to ICU	N/A	12 (20%)	15 (52%)	N/A	N/A	<0.05
LOS in ICU (days)	N/A	N/A	17	N/A	N/A	N/A
Death	N/A	8 (13.5%)	12 (41.5%)	N/A	N/A	<0.05

*p* values were calculated as follows: continuous variables by Kruskal–Wallis test, nonparametric, adjusted *p* value for multiple comparison and by Mann–Whitney test, nonparametric test, adjusted *p* value for two groups. Categorial variables by contingency, Fisher’s exact test. *p* value < 0.05 is considered statistically significant. LOS, length of stay, ns: not significant, N/A: not applicable.

## Data Availability

Raw FASTQ data discussed in this publication have been deposited in NCBI’s Sequence Read Archive under BioProject accession PRJNA901461. Count data were deposited to NCBI’s Gene Expression Omnibus [70] and are accessible through GEO Series accession number GSE217948 (https://www.ncbi.nlm.nih.gov/geo/query/acc.cgi?acc=GSE217948, accessed on 10 December 2022). Details can be found in Supplement Datasheet S4.

## Supplementary Materials

The following supporting information can be downloaded at: https://www.mdpi.com/article/10.3390/ijms24032524/s1. References [67,68,69] are cited in the supplementary materials.
