# Supplementary material for: Pathway and Network Analyses Identify Growth Factor Signaling and MMP9 as Potential Mediators of Mitochondrial Dysfunction in Severe COVID-19
Source: Int J Mol Sci. 2023 Jan 28;24(3):2524. doi: 10.3390/ijms24032524 (PMC9917147; doi:10.3390/ijms24032524)
Supplement: Supplementary file 1 [file ijms-24-02524-s001.zip › Supplement Figures_kls_100123_1.pdf]

Supplementary Figure S1. Principal Component Analysis (PCA) of all coding genes. PCA plot of PC1 and PC2 showing levels of healthy controls (HC), mild/moderate (MldMod) and severe (Svre) COVID-19 cases by different colours. PCA was generated from normalized rlog data. Note that this analysis and the results are very similar to our previous study ("Blood transcriptome responses in patients correlate with severity of COVID-19 disease" 10.3389/fimmu.2022.1043219. Accepted but not yet available at PubMed) with a slightly different sample selection (here, we did not include multiple samples from the same patient).

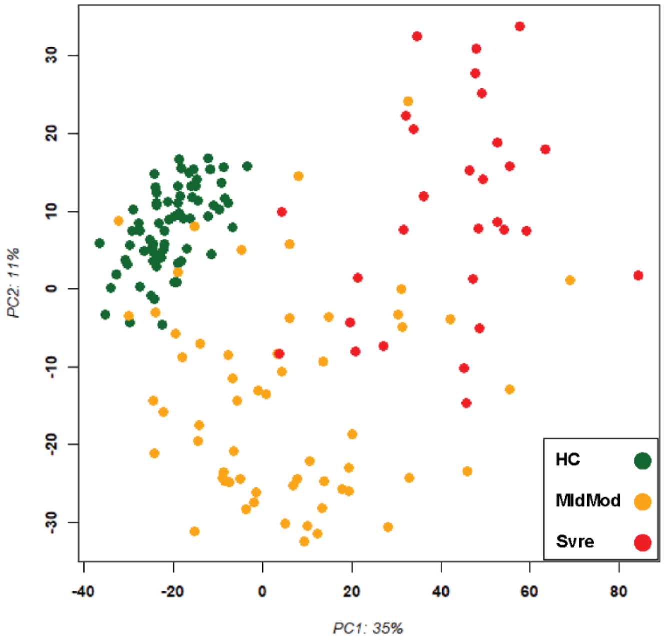

Supplementary Figure S2. Deconvolution analysis of all coding genes. Mean values for mild/moderate, severe, and healthy control groups were calculated and subjected to a deconvolution analysis. Deconvolution was performed with the package immunedeconv (version 2.0.4) [1] using the method mcp_counter [2]. Y-axis: severity categories, X-axis: scores from mcp_counter analysis for the different cell populations. HC: healthy controls; mld_mod: mild and moderate; svre: severe. This analysis revealed a slight increase in endothelial cell and neutrophil in the mild/moderate cases compared to the healthy controls. A much stronger increase was observed in the severe cases. Conversely, for B, T, and dendritic cells, a slight decrease was observed for the mild/moderate cases compared to healthy controls whereas a strong decrease was evident in the severe cases compared to either healthy controls or mild/moderate cases. For macrophage/monocyte and NK cells, an increase in their relative abundance was observed in the mild/moderate cases compared to the healthy controls and a strong reduction in the severe cases compared to both the health controls and the mild/moderate cases. Note that this analysis and the results are very similar to our previous study ("Blood transcriptome responses in patients correlate with severity of COVID-19 disease" 10.3389/fimmu.2022.1043219. Accepted but not yet available at PubMed) with a slightly different sample selection (here, we did not include multiple samples from the same patient).

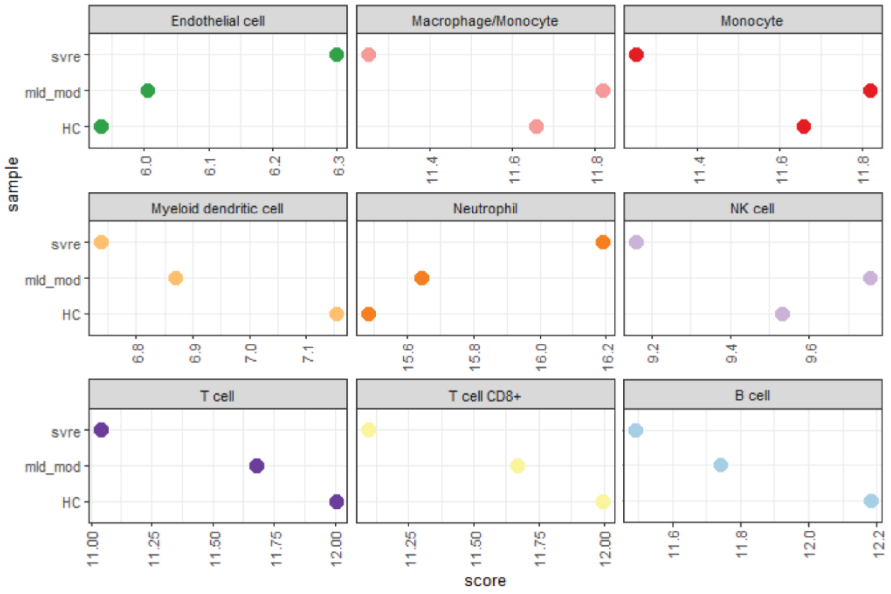

Supplementary Figure S3. Functional analysis of mitochondria specific DEGs. Functional analysis using GO term enrichment for DEGs from the contrast of mild/moderate versus severe cases. Pathway association analysis was performed with the package clusterProfiler [3] **(A)** up-regulated (higher in severe), **(B)** down-regulated (higher in mild/moderate).

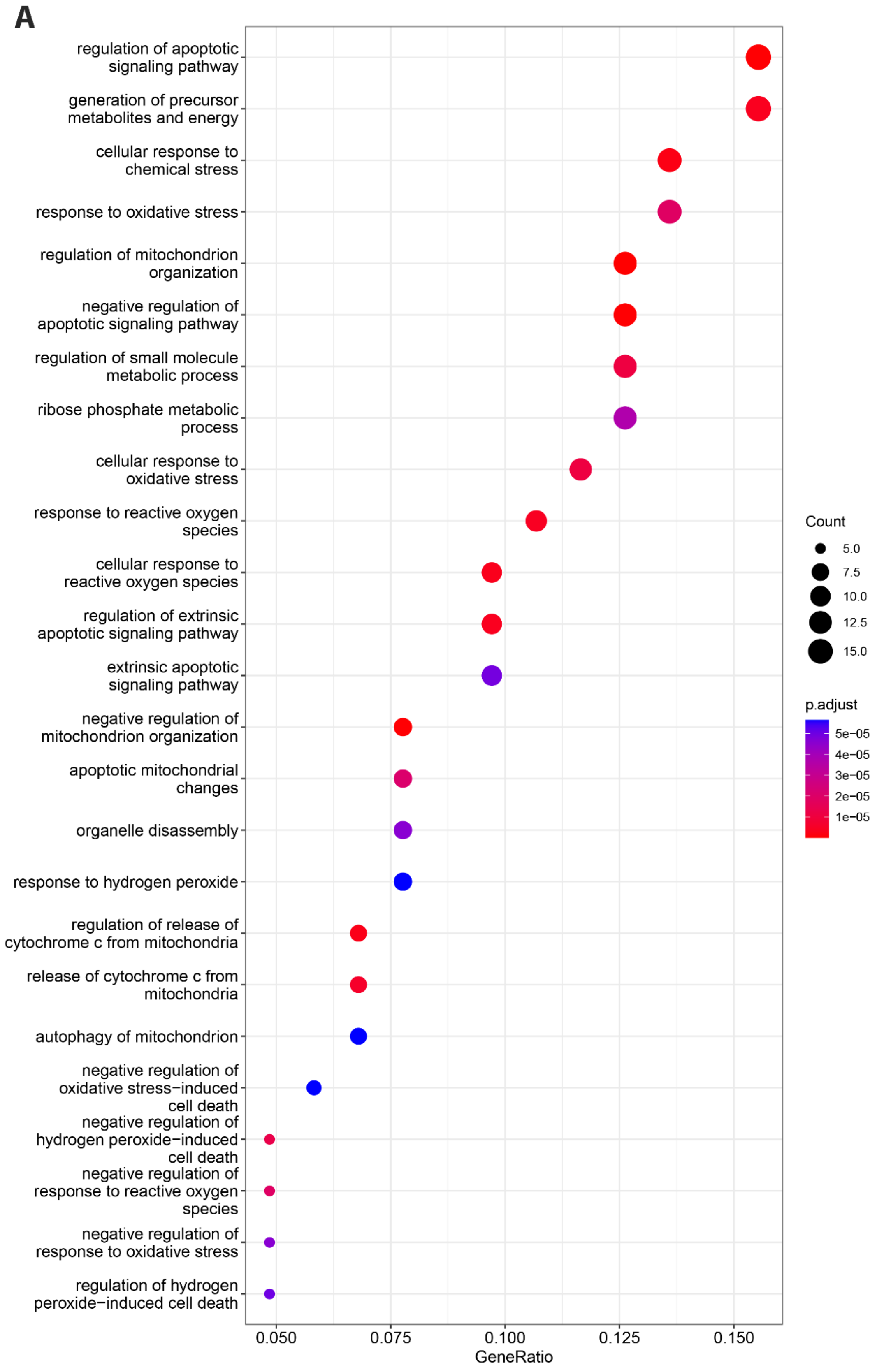

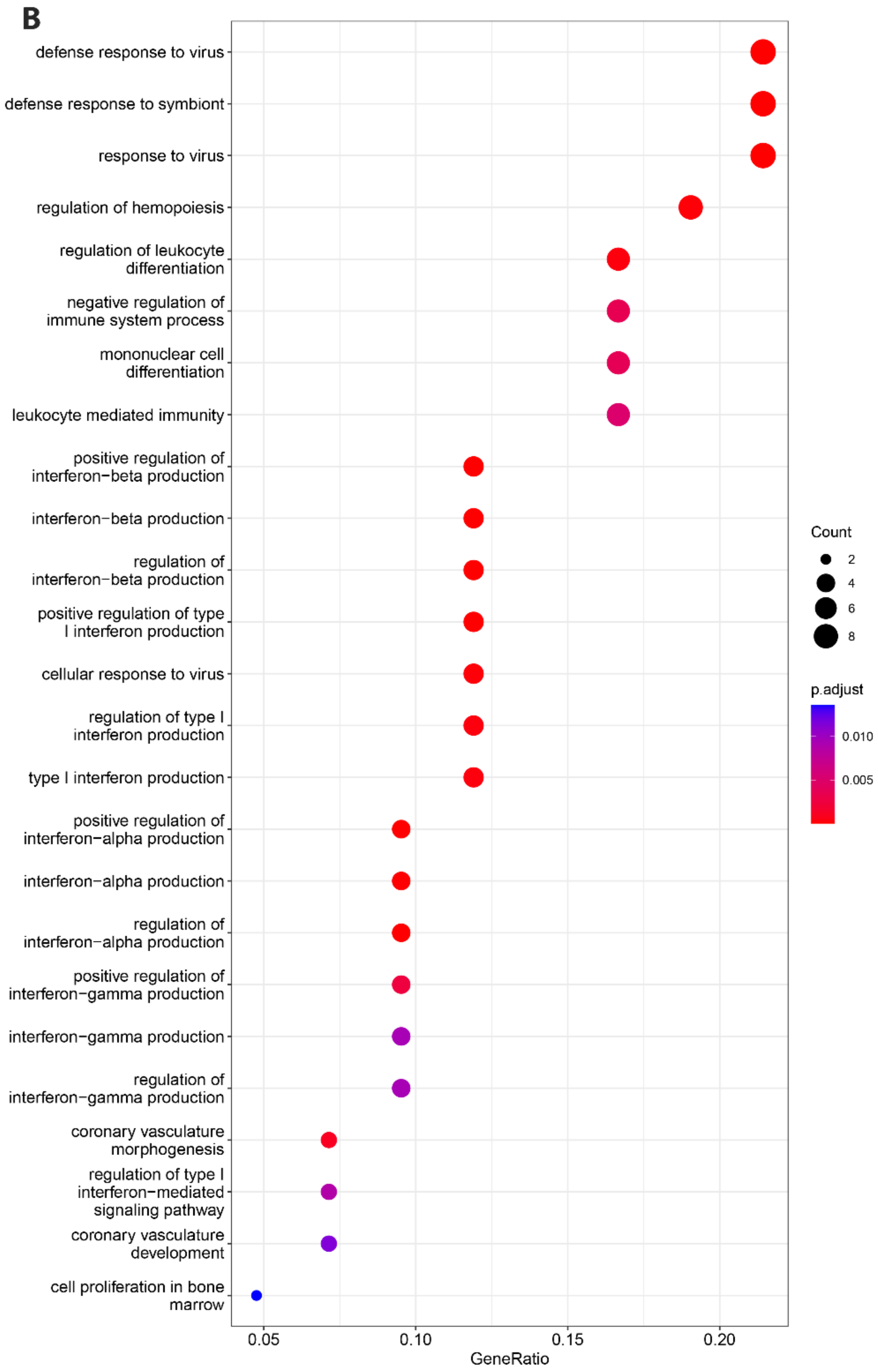
B
defense response to virus
defense response to symbiont
response to virus
regulation of hemopoiesis
regulation of leukocyte differentiation
negative regulation of immune system process
mononuclear cell differentiation
leukocyte mediated immunity
positive regulation of interferon−beta production
interferon−beta production
regulation of interferon−beta production
positive regulation of type I interferon production
cellular response to virus
regulation of type I interferon production
type I interferon production
positive regulation of interferon−alpha production
interferon−alpha production
regulation of interferon−alpha production
positive regulation of interferon−gamma production
interferon−gamma production
regulation of interferon−gamma production
coronary vasculature morphogenesis
regulation of type I interferon−mediated signaling pathway
coronary vasculature development
cell proliferation in bone marrow
0.05
0.10
0.15
0.20
GeneRatio
Count
2
4
6
8
p.adjust
0.010
0.005

Supplementary Figure S4. Metacore network analysis of genes from the enriched pathways. (A) Bar diagram showing Log_2 Fold Change (LFC) of the genes that contribute to enrichment of the top 10 pathways from Figure 3D (Svre vs MldMod_UP). Network analysis of the genes from six of the ten pathways (highlighted in red) were shown in **(B)** cell adhesion ECM remodelling **(C)** androgen signalling in HCC **(D)** IL-b and endothelin-1-induced fibroblast/myofibroblast migration and ECM production in asthmatic airways (**E)** stromal-epithelial interaction in prostate cancer **(F)** cell cycle chromosome condensation in prometaphase **(G)** DNA damage ATM/ATR regulation of G2/M checkpoint: nuclear signalling. **(H)** Bar diagram showing Log_2 Fold Change (LFC) of the genes that contribute to enrichment of the top 10 pathways from Figure 3E (Svre vs MldMod_DOWN). Network analysis of the genes from four of the ten pathways (highlighted in blue) were shown in **(I)** COVID-19: immune dysregulation **(J)** COVID-19: regulation of antiviral response by SARS-CoV-2 **(K)** glomerular injury in lupus nephritis (**L)** dual function of Treg cells in cancer **(M)** MAPK-independent proliferation of normal and asthmatic smooth muscle cells **(N)** $CD8^+$ Tc1 cells in allergic contact dermatitis. Red and blue circles denote upregulated and downregulated genes from the respective enriched pathway. The coloured solid line with arrows represents activation (green), inhibition (red) and unspecified (grey) effects between the two genes. Bold light green lines represent well-known canonical pathways. Abbreviations: E stands for extracellular space; PM stands for plasma membrane; C stands for cytosol; N stands for nucleus.

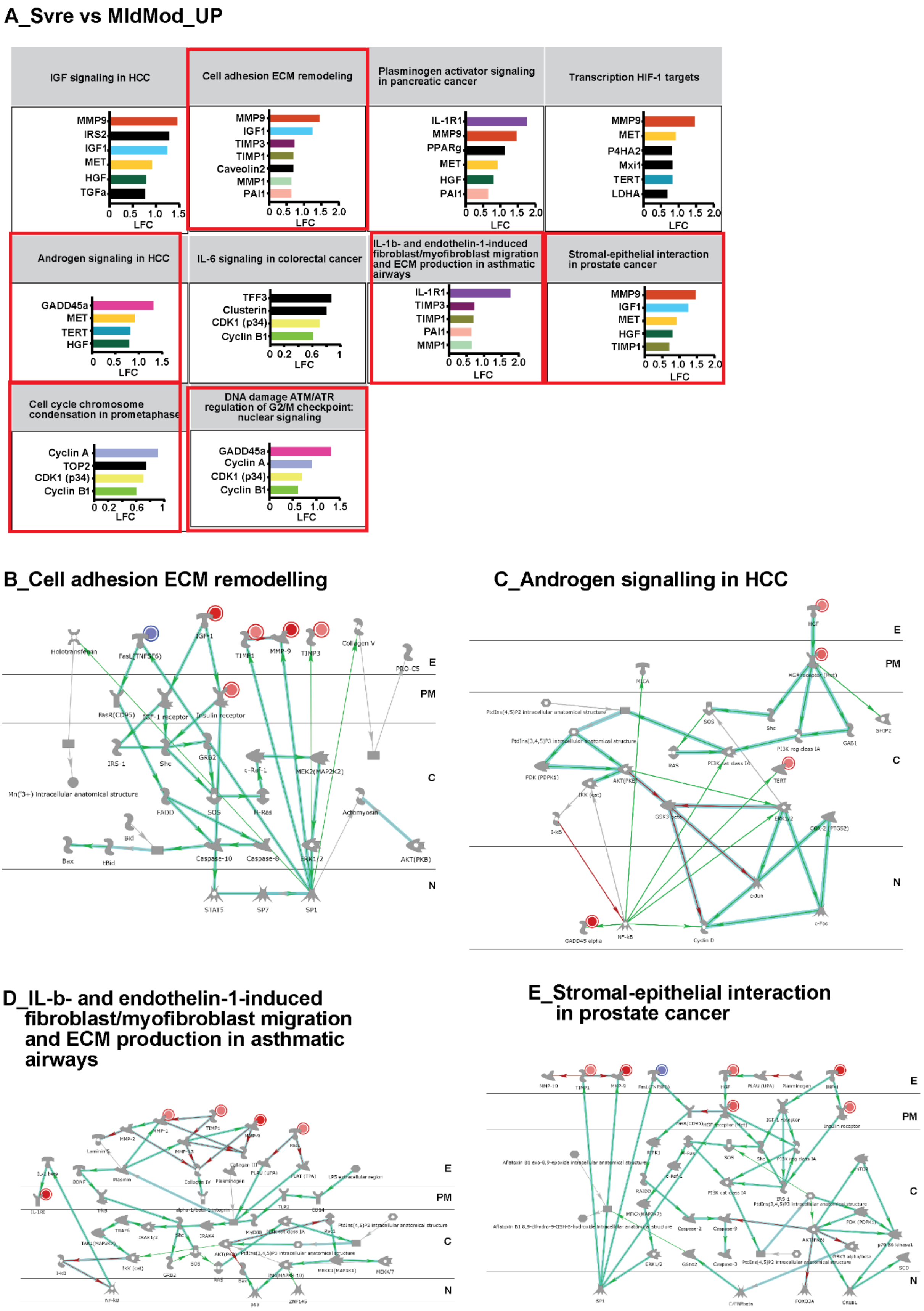
A_Svre vs MldMod_UP
IGF signaling in HCC
MMP9
IRS2
IGF1
MET
HGF
TGFa
0 0.5 1.0 1.5
LFC
Cell adhesion ECM remodeling
MMP9
IGF1
TIMP3
TIMP1
Caveolin2
MMP1
PAI1
0 0.5 1.0 1.5 2.0
LFC
Plasminogen activator signaling in pancreatic cancer
IL-1R1
MMP9
PPARg
MET
HGF
PAI1
0 0.5 1.0 1.5 2.0
LFC
Transcription HIF-1 targets
MMP9
MET
P4HA2
Mxi1
TERT
LDHA
0 0.5 1.0 1.5 2.0
LFC
Androgen signaling in HCC
GADD45a
MET
TERT
HGF
0 0.5 1.0 1.5
LFC
IL-6 signaling in colorectal cancer
TFF3
Clusterin
CDK1 (p34)
Cyclin B1
0 0.2 0.6 1
LFC
IL-1b- and endothelin-1-induced fibroblast/myofibroblast migration and ECM production in asthmatic airways
IL-1R1
TIMP3
TIMP1
PAI1
MMP1
0 0.5 1.0 1.5 2.0
LFC
Stromal-epithelial interaction in prostate cancer
MMP9
IGF1
MET
HGF
TIMP1
0 0.5 1.0 1.5 2.0
LFC
Cell cycle chromosome condensation in prometaphase
Cyclin A
TOP2
CDK1 (p34)
Cyclin B1
0 0.2 0.6 1
LFC
DNA damage ATM/ATR regulation of G2/M checkpoint: nuclear signaling
GADD45a
Cyclin A
CDK1 (p34)
Cyclin B1
0 0.5 1.0 1.5
LFC
B_Cell adhesion ECM remodelling
E
PM
C
N
C_Androgen signalling in HCC
E
PM
C
N
D_IL-b- and endothelin-1-induced fibroblast/myofibroblast migration and ECM production in asthmatic airways
E
PM
C
N
E_Stromal-epithelial interaction in prostate cancer
E
PM
C
N

F_Cell cycle chromosome condensation in prometaphase

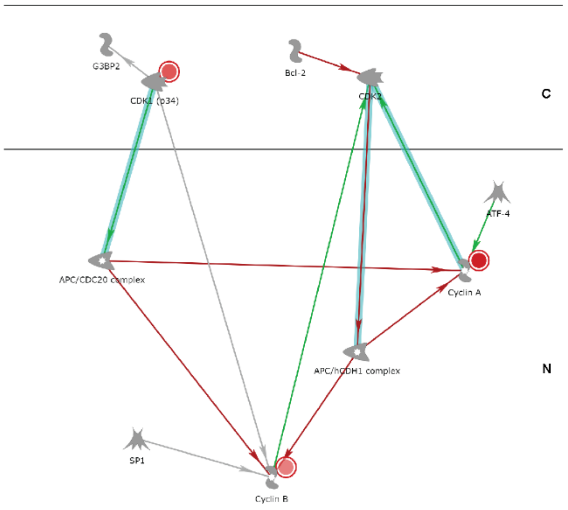

G_DNA damage ATM/ATR regulation of G2/M checkpoint: nuclear signalling

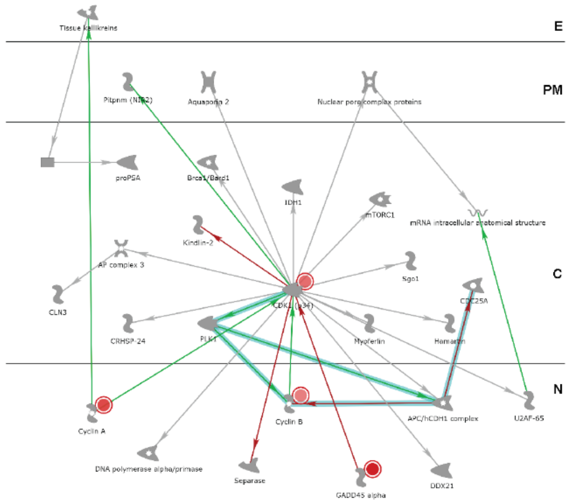

H_Svre vs MldMod_DOWN

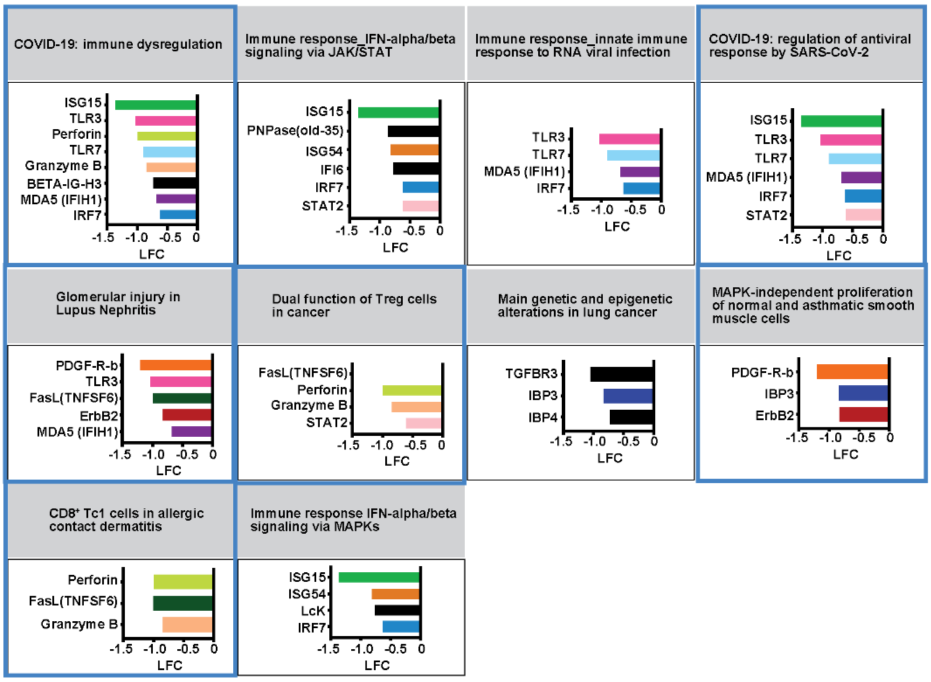

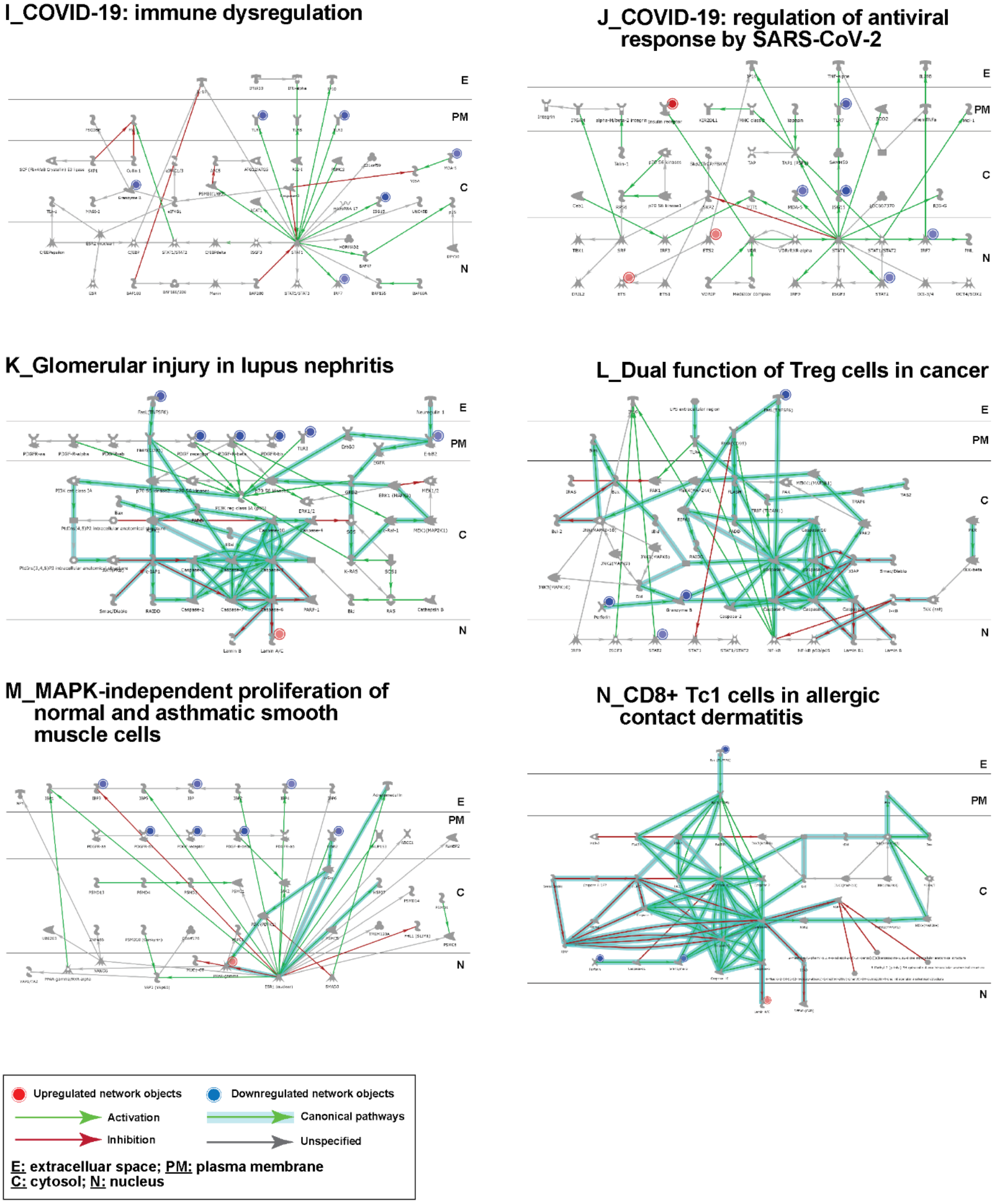
I_COVID-19: immune dysregulation
J_COVID-19: regulation of antiviral response by SARS-CoV-2
K_Glomerular injury in lupus nephritis
L_Dual function of Treg cells in cancer
M_MAPK-independent proliferation of normal and asthmatic smooth muscle cells
N_CD8+ Tc1 cells in allergic contact dermatitis
E
PM
C
N
Upregulated network objects
Downregulated network objects
Activation
Canonical pathways
Inhibition
Unspecified
E: extracellular space; PM: plasma membrane
C: cytosol; N: nucleus

References

1. Finotello, F., C. Mayer, C. Plattner, G. Laschober, D. Rieder, H. Hackl, A. Krogsdam, Z. Loncova, W. Posch, D. Wilflingseder, S. Sopper, M. Ijsselsteijn, T. P. Brouwer, D. Johnson, Y. Xu, Y. Wang, M. E. Sanders, M. V. Estrada, P. Ericsson-Gonzalez, P. Charoentong, J. Balko, Nfdcc de Miranda, and Z. Trajanoski. "Molecular and Pharmacological Modulators of the Tumor Immune Contexture Revealed by Deconvolution of Rna-Seq Data." *Genome Med* 11, no. 1 (2019): 34.
2. Becht, E., N. A. Giraldo, L. Lacroix, B. Buttard, N. Elarouci, F. Petitprez, J. Selves, P. Laurent-Puig, C. Sautès-Fridman, W. H. Fridman, and A. de Reyniès. "Estimating The population Abundance of Tissue-Infiltrating Immune and Stromal Cell Populations Using Gene Expression." *Genome Biol* 17, no. 1 (2016): 218.
3. Yu, G., L. G. Wang, Y. Han, and Q. Y. He. "Clusterprofiler: An R Package for Comparing Biological Themes among Gene Clusters." *Omics* 16, no. 5 (2012): 284-7.
